# Crosstalk of the Caspase Family and Mammalian Target of Rapamycin Signaling

**DOI:** 10.3390/ijms22020817

**Published:** 2021-01-15

**Authors:** Junfang Yan, Yi Xie, Jing Si, Lu Gan, Hongyan Li, Chao Sun, Cuixia Di, Jinhua Zhang, Guomin Huang, Xuetian Zhang, Hong Zhang

**Affiliations:** 1 Institute of Modern Physics, Chinese Academy of Sciences, 509 Nanchang Road, Lanzhou 730000, China; yanjf@impcas.ac.cn (J.Y.); sijing@impcas.ac.cn (J.S.); ganl@impcas.ac.cn (L.G.); lihy@impcas.ac.cn (H.L.); sunchao@impcas.ac.cn (C.S.); dicx@impcas.ac.cn (C.D.); zhangjinhua@impcas.ac.cn (J.Z.); huangguomin@impcas.ac.cn (G.H.); zhangxuetian@impcas.ac.cn (X.Z.); 2 Advanced Energy Science and Technology Guangdong Laboratory, Huizhou 516029, China; 3 Key Laboratory of Heavy Ion Radiation Biology and Medicine of Chinese Academy of Sciences, Lanzhou 730000, China; 4 Key Laboratory of Heavy Ion Radiation Medicine of Gansu Province, Lanzhou 730000, China; 5 College of Life Sciences, University of Chinese Academy of Sciences, Beijing 101408, China

**Keywords:** the caspase family, mTOR signaling, cell fate, interplay

## Abstract

Cell can integrate the caspase family and mammalian target of rapamycin (mTOR) signaling in response to cellular stress triggered by environment. It is necessary here to elucidate the direct response and interaction mechanism between the two signaling pathways in regulating cell survival and determining cell fate under cellular stress. Members of the caspase family are crucial regulators of inflammation, endoplasmic reticulum stress response and apoptosis. mTOR signaling is known to mediate cell growth, nutrition and metabolism. For instance, over-nutrition can cause the hyperactivation of mTOR signaling, which is associated with diabetes. Nutrition deprivation can inhibit mTOR signaling via SH3 domain-binding protein 4. It is striking that Ras GTPase-activating protein 1 is found to mediate cell survival in a caspase-dependent manner against increasing cellular stress, which describes a new model of apoptosis. The components of mTOR signaling-raptor can be cleaved by caspases to control cell growth. In addition, mTOR is identified to coordinate the defense process of the immune system by suppressing the vitality of caspase-1 or regulating other interferon regulatory factors. The present review discusses the roles of the caspase family or mTOR pathway against cellular stress and generalizes their interplay mechanism in cell fate determination.

## 1. Introduction

The optimal growth of cell is closely connected to the stress response. It is necessary to integrate the pro-growth signaling and stress response pathways to maintain the balance of growth and death. Once this balance is destroyed, malignant proliferation or cell death will occur and the organism will develop diseases such as tumors. Massive and intense external environmental stimuli can change intracellular homeostasis, causing various cellular stresses such as inflammation, oxygen stress, energy and nutrition metabolism stress and endoplasmic reticulum (ER) stress [1]. Therefore, according to different stress levels and models, different response mechanisms and cell protective strategies will be carried out accordingly. Caspase family members are powerful function proteins that respond to cellular stress including cell death signals, inflammation and ER stress [2,3,4]. Caspases-mediated apoptosis is a kind of suicide protective measure initiated by gene regulation, which aims at eliminating redundant, damaged or effete cells under stress [5]. Mammalian target of rapamycin (mTOR) signaling transduction constitutes a complex and huge cascade network, regulating protein synthesis, cell differentiation, the cell cycle, cell growth and metabolism [6,7]. Existing research has identified the critical regulation mechanism between mTOR signaling and the caspase family [8,9]. In this review, we focus on the cross talk of the two pathways in the determination of cell life and death.

## 2. The Caspase Family

**Figure 1:**
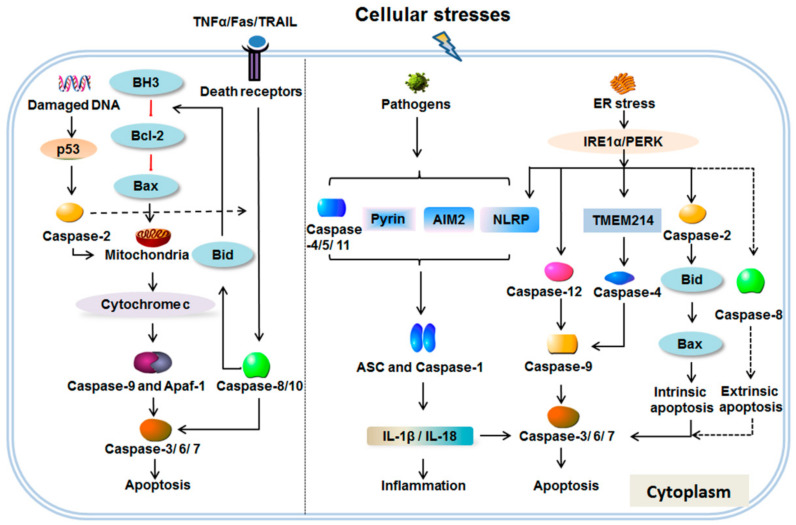
Apoptosis, inflammation and endoplasmic reticulum (ER) stress regulated by the caspase family. Stress stimulation such as DNA damage can trigger intrinsic apoptosis, in which releasing cytochrome c activates the complex of apoptosis-activating factor 1 (Apaf-1) and caspase-9, initiating the auto-activation of caspase-9. The binding of cell surface death ligand and receptors induces extrinsic apoptosis, and caspase-8 or caspase-10 are the initiator caspases. Caspase-3, -6 and -7 execute apoptosis effector of both apoptosis pathways to catalyze a variety of key cellular substrates. Death stimulations restrain anti-apoptotic members of the Bcl-2 subfamily through the pro-apoptotic factors BH3 subfamily and then activate the pro-apoptotic factors Bax subfamily, functioning as the co-factor of caspases. The central member of the caspases in response to the inflammation is caspase-1. Inflammasome sensors (The nucleotide-binding domain and leucine-rich repeat containing (NLR) family, the HIN200 family member absent in melanoma 2 (AIM2) and the TRIM family member Pyrin) identify the inflammatory irritants such as pathogens and activate caspase-1 by forming a complex with the adaptor protein apoptosis associated speck like protein containing a CARD (ASC) and caspase-1 to promote the maturation and secretion of IL-1β and IL-18 in the canonical inflammasome pathway. Caspase-4, -5 and -11 respond to lipopolysaccharide arising from Gram-negative bacterial infection with the non-canonical inflammasome pathway. Finally, severe inflammations lead to apoptosis. ER stress response is mediated by caspase-4, -5, -8 and -12 with different mechanisms to induce inflammation and apoptosis. Inositol-requiring enzyme 1 (IRE1α) and PKR-like ER kinase (PERK) are the transmembrane sensors of ER. The Human transmembrane protein 214 (TMEM214) locates at the upstream of caspase-4.

Caspases, a family of cysteine proteases, contain 14 identified members. The protease properties enable them to cleave a series of substrates at specific cleaved sites [10]. According to the phylogenetic analysis, this gene family is divided into two major subfamilies: the caspase-1 (interleukin-1β converting enzyme, ICE) subfamily and the product of cell-death abnormal gene-3 (CED-3) subfamily. The ICE subfamily containing caspase-1, -4, -5, -11, -12 and -13 acts as the regulator of inflammation and ER stress-specific apoptosis [4,11,12]. The second subfamily is mainly activated in the apoptosis process and classified into the caspase-3 subclass including caspase-3, -6 and -7 as executors and the caspase-9 subclass including caspase-2, -8, -9 and-10 as initiators on account of their role in apoptosis [13,14]. Caspases constitute a protease cascade that can rapidly amplify cell death and inflammation response (Figure 1).

### 2.1. Apoptosis Response

Extensive research has shown that apoptosis is initiated by the initiators receiving death receptor, DNA damage, mitochondrial damage and ER stress, which activates caspase precursors by upstream driver or auto-processing to process substrates. The proximity model elaborates the auto-activation of caspase-2, -8, -9 and -10, which typically occurs when they are close to or combine with each other [15]. Active initiators further process precursors of the effector caspases. Caspase-3, -6 and -7 contribute to coordinating the execution phase of apoptosis by cleaving multiple downstream molecules to induce cell death [16].

Classic apoptotic pathways have been extensively elaborated, which includes the intrinsic apoptosis caused by releasing cytochrome c from mitochondria into the cytosol and the extrinsic apoptosis mediated by cell surface death ligand and receptors (tumor necrosis factor-α (TNFα); Fas and TNF related apoptosis inducing ligand (TRAIL)) (Figure 1) [17]. The Bcl-2 subfamily is the co-factor of caspases in controlling apoptosis. Death stimulations such as oncogene activation, DNA damage, lacking of cell growth factor or cell hypoxia can inhibit the anti-apoptotic members of the Bcl-2 subfamily (Bcl-2, Bcl-XL, Bcl-w, Al and Mcl-1) through pro-apoptotic factors of the BH3 subfamily (Bik, Bim_L_, Bad and Bid) and then activate pro-apoptotic factors of the Bax subfamily (Bax and Bak). This process increases the permeability of mitochondria and causes the release of cytochrome c from mitochondria [18]. The release of cytochrome c activates the complex of apoptosis-activating factor 1 (Apaf-1) and pro-caspase-9 via respective NH2-terminal CED-3-homologous domains, initiating the auto-activation of caspase-9, which in turn activates pro-caspase-3, -6 and -7 [19]. In addition to cytochrome c, Smac is another pro-apoptotic factor in mitochondria that works by blocking the activation of X-linked inhibitor of apoptosis protein whose BIR domains are contributed to the blocking activation of caspase-3 and -7 [20]. At the same time, the BIR3 domain of X-linked inhibitor of apoptosis protein can restrain the caspase-9/Apaf-1 complex by binding to the amino terminus of the linker peptide on the small subunit of caspase-9 [21].

The long history of the extrinsic apoptosis field has indicated that death receptor-mediated cell death is driven by a caspase cascade in which the caspase-1-like proteases send death signals from caspase-8 proteases to caspase-3-like effectors. The death domains of TNFα, TRAIL or Fas ligand recruit the death domain adaptor proteins, which in turn results in the binding of death-inducing signaling complex (DISC) and pro-caspase-8 [22,23]. Pro-caspase-8 is activated in the DISC and its mature form is released, initiating a cascade of events that lead to apoptosis. Similarly, caspase-10 is another death receptor initiator caspase [24]. Despite intrinsic and extrinsic apoptosis responding to different death stimulations, the two apoptotic pathways can feedback with each other. For instance, Bid is cleaved by caspase-8 to truncate Bid. Active Bid is transferred from cytosol to the mitochondrial membrane where it interacts with the anti-apoptotic Bcl-2 family and results in further release of cytochrome c [25]. The role of caspase-2 in a specific apoptotic pathway is controversial, and it has been reported to mediate the death receptor-mediated apoptosis [13]. In this process, RIP-associated ICH1/CED3-homologous protein with death domain is the adaptor protein of caspase-2 pro-domain in a p53-induced protein with death domain (PIDD)-osome-independent manner [26]. To finish the proteolytic cascade of apoptosis mediated by death receptors, the cytochrome c-activated caspase-9/caspase-3 pathway activates pro-caspase-2 [27], or caspase-2 handles pro-caspase-8 monomers via TRAIL engagement through protein kinase CK2 phosphorylating pro-caspase-2 directly at serine-157 [26]. Proven significance of caspase 2 has been shown in response to the cell death induced by damaged DNA [28]. Caspase-2 is regulated by p53 in response to the DNA damage agents [29]. As long as DNA damage occurs, p53-induced protein with death domain (PIDD) produces PIDD-C and PIDD-CC fragments via post-translational auto-catalysis at Ser 446 and Ser 588 cleavage sites. PIDD-C owning a death domain could mediate the nuclear factor kappa-B (NF-κB) signal through the auto-proteolysis mechanism at Ser 588, and then form a complex with caspase-2 to trigger apoptosis via the intrinsic apoptosis pathway [30]. In an early phase of apoptosis, cytochrome c is released in the intact nuclear/cytoplasmic barrier. Caspase-2 as a protease imports into the nuclear and is regulated by two nuclear localization signals in the pro-domain. Until the late phase of apoptosis, caspase-2 acts in the cytoplasm [31].

The crucial function of caspase-3 is better understood as it is frequently activated by abundant stimulus signals, acting as the integrator of multiple apoptotic pathways and catalyzing a variety of key cellular substrates. Events that caspase-3 engaging on include chromatin margination, cell division, nuclear condensation and DNA fragmentation [16]. Although caspase-3 acts as the most important executioner, the absence of caspase-3 or homozygous deletion of caspase-3 can also lead to cell death. This indicates that the caspase-7-mediated pathway is an alternative and compensatory method of functional caspase-3 deletion [32]. The role of caspase-6 in nuclear disassembly is demonstrated by the caspase-6 inhibitor z-VEID-fmk. Z-VEID-fmk could prevent caspase-6 from cleaving lamin A. This cleavage is necessary for the complete condensation of chromosomal DNA in the process of apoptosis [33]. The function inhibition of caspase-3 is particularly important in cell cycle regulation. The upregulation of survivin can inhibit the activation of caspase-3 and caspase-7 in the G_2_/M phase and ensure normal cell division [34].

### 2.2. Inflammation and Endoplasmic Reticulum (ER) Stress Response

A number of human heritable and acquired diseases have strong associations with dysregulated inflammatory events [35]. Based on the functional models and type of stimulators, inflammasomes are divided into canonical and non-canonical inflammasome pathways. Caspase-1 is the key caspase member in the canonical inflammasome pathway which responds to extensive environmental pathogens and invaders [36]. In contrast to caspase-1, caspase-4, -5 and -11 act mainly in response to lipopolysaccharide coming from Gram-negative bacterial infection with the non-canonical inflammasome pathway. Different inflammatory stimulators enlist the relevant inflammasome sensors to initiate inflammation (Figure 1). A recent review has generalized the type of inflammasome sensors according to the inflammatory stimulators [36]. The nucleotide-binding domain and leucine-rich repeat containing (NLR) family, the HIN200 family member absent in melanoma 2 (AIM2) and the TRIM family member Pyrin execute the function of inflammasome sensors. For example, NLRP3 extensively recognizes pathogen-associated molecular patterns, danger-associated molecular patterns and protein aggregates and AIM2 mainly responds to dsDNA. In the canonical inflammasome pathway, the key components of inflammasome are comprised of inflammasome sensors, the adaptor protein and caspase-1. The adaptors of caspase-1 include apoptosis associated speck like protein containing a CARD (ASC), interleukin betaconverting enzyme activating factor (Ipaf) and receptor-interacting protein 2 (RIP2) [37]. Related research has demonstrated that ASC deficiency can inactivate caspase-1 driven by ATP stimulation and pathogens. The lack of Ipaf and RIP2 were not shown to affect the activation of caspase-1 induced by pathogens. This result indicates that ASC is the primary adaptor of caspase-1 [37]. Ipaf and RIP2 contribute to the specific inflammatory stimulus. For instance, Ipaf recognizes cytosolic dsDNA directly and regulates the activation of caspase-1 and inflammasome formation, and its CARD domain interacts with the CARD domain of pro-caspase-1 [38]. Moreover, Caspase-1 integrates information and executes its pro-inflammatory function via the proteolysis of pro-IL-1β and IL-18. Subsequently, severe inflammation triggers caspase-3, -6 and -7-dependent cell death [36,39,40]. In addition, there are different response mechanisms in the response of caspase-4, -5 and -11 to Gram-negative bacterial infection. The obstruction of caspase-5 decreases the protein expression of caspase-1 and inhibits TNF-α-induced IL-8 and monocyte chemotactic protein 1 after pro-inflammatory stimuli [41]. Research shows that caspase-11 is the upstream regulator of caspase-1 at the TIR-domain-containing adaptor-inducing interferon-β/IFN/caspase-11 axis in all Gram-negative bacterial infections [42]. The pyroptosis induced by the activation of caspase-4 in response to cytosolic lipopolysaccharide relies on the maturation of IL-1β triggered by the NLRP3/ASC/caspase-1 complex [43].

The correlation between the upregulation of inflammation and ER stress markers in human steatohepatitis liver biopsies indicates that ER stress is an important factor involved in the production of inflammatory and apoptosis [44] (Figure 1). The activation of transmembrane sensors such as inositol-requiring enzyme 1 (IRE1α) and PKR-like ER kinase (PERK) under ER stress result in the NLRP3 inflammasome activation by inducing the overexpression of C/EBP homologous protein (CHOP) [45,46]. The ER stress inducer can enhance caspase-5 and -1 activity [41]. The significance of caspase-5 in the regulation of inflammation has again been proven. Among the members of the caspase family, caspase-2, -4 and -12 are found to be localized at the ER and involved in the response of ER stress [11,47,48]. The ER stress induced by misfolded proteins leads to the activation of Bax and Bak. This process can be enhanced by the activation of the BH3-only protein Bid cleaved by pro-caspase-2 [49]. However, other research suggests that ER stress-induced apoptosis does not require caspase-2 activation [50]. Different inflammatory inducers and ER stress lead to caspase-4 activation [51]. For example, small interfering RNA of caspase-4 suppresses apoptosis and directly activates caspase-9 at Asp-315 upon ER stress [48,52]. Human transmembrane protein 214 (TMEM214) is found to be the upstream regulator of caspase-4 [53]. Caspase-12 is highly homologous with caspase-1 (39%) [14] and plays a central role in mediating ER homeostasis [54]. ER stress can trigger apoptosis via caspase-12 activating cytochrome c-independent caspase-9 [14]. The mechanism of pro-caspase-12 activation is complex and needs deeper investigation. There are several models for the activation of pro-caspase-12. One model points out that once cells undergo ER stress, pro-caspase-12 recognizes adaptor molecule TNF receptor associated factor 2 and interacts with Ire1α. Then pro-caspase-12 is detached from TNF receptor associated factor 2, and then homodimerization and auto-activation occurs [54]. Another mechanism is that the release of Ca^2+^ because of ER stress activates m-calpain and cleaves the pro-domain of caspase-12 [54]. Moreover, the role of another caspase member caspase-8 in mediating ER stress is still controversial. Glab et al. found that death receptor 5 or caspase-8 knockout cell lines obtained by CRISPR technology were still sensitive to ER stress inducers. They concluded that ER stress-induced apoptosis is caspase-8 or death receptor 5-independent [55]. However, Cristina Munóz-Pinedo et al. disputed the result of Glab et al. They analyzed the data and suggested that death receptor 5 and caspase-8 were essential in response to ER stress in many models which depends on cell types [56]. Similarly, Mable Lam further confirmed that the knockout of caspase-8 or death receptor 5 significantly inhibited the apoptosis process induced by the ER stress inducer thapsigargin using the cell lines that Glab et al. provided and the knockdown of caspase-8 or death receptor 5 via siRNA came to the same conclusion [57]. Other research has demonstrated that in breast epithelial cells with ERBB2 oncogene mutation, hyperactivation of the PERK/ATF4/CHOP pathway leads to the upregulation of pro-apoptotic cell surface receptor TRAIL-receptor 2 expression while undergoing ER stress, following a formation of a DISC complex composed of TRAIL-receptor 2, Fas-associating protein with a novel death domain and pro-caspase-8, resulting in the activation of caspase-8 in response to the ER stress-induced apoptosis [58]. These indicate that the caspase-8 in response to ER stress depends on the types of cells and ER stress inducers. Although caspase-2, -4, -5, -8 and -12 play important mediating roles for ER stress response, the specific regulatory mechanism remains to be further studied.

## 3. The Multiple Functions of mTOR Signaling in Cells

mTOR is a serine (Ser)/threonine (Thr) kinase and belongs to the phosphoinositide 3-kinase-related protein kinase family [59]. mTORC1 and mTORC2 are the two distinct multiprotein complexes of mTOR in function and biochemistry. The mTORC1 complex consists of mTOR, GβL and raptor. The inhibitor rapamycin can bind with raptor via FKBP12, perturbing the raptor-mTOR interaction [60]. The mTORC2 complex, also named PDK2, is comprised of mTOR, GβL and rictor. Rictor is a rapamycin-insensitive companion and is less conserved than other components [61]. The activation of mTORC1 modulates the processing of protein synthesis, nutrient metabolism, ribosome biogenesis and autophagy [62,63,64]. The function of the mTORC2 complex mainly focuses on cytoskeleton reorganization and cell growth [65,66]. According to different functional requirements, the mTOR complexes can be detected in intracellular organelles such as the nucleus, lysosome, mitochondria and ER [67]. They rely on the Ras/phosphatidylinositol 3-kinase (PI3K)/Akt/mTOR pathway to function as a crucial modulator in coordinating the balance between cell growth and cell death in response to growth factors, nutritional status and other stress signals.

### 3.1. mTOR Signaling Responds to Growth Factors

The activation of mTORC1 via the Ras/PI3K/Akt/mTOR pathway is the central event in the response to growth factor stimulation [68]. As a crucial biochemical network, the Ras/PI3K/Akt/mTOR pathway is coordinated with the Ras/Raf/Mitogen-activated protein kinase kinase (MEK)/extracellular signal-regulated kinase (ERK) pathway to control the growth of organisms through the phosphorylation and dephosphorylation mechanism by specific kinases [69]. Growth factors such as insulin and insulin-like growth factor recognize receptor tyrosine kinases (RTKs) and trigger the kinases cascade by activating two key signal-transduction molecules: the small GTPase Ras and the lipid kinase PI3K [70]. The binding of ligands and RTKs leads to the dimerization and activation of RTKs, which in turn phosphorylates tyrosine sites of cytoplasmic tails and recruits SRC homology domain 2 (SH2) or phosphotyrosine binding domains of adaptor proteins such as growth factor receptor-bound protein 2 (GRB2) and insulin receptor substrate (IRS) [70]. The IRS family has been identified as an insulin receptor substrate [71]. Son of seven less homolog protein (SOS), a Ras guanine-nucleotide-exchange factor (GEF), is subsequently activated and stimulates the exchange of GDP for GTP on Ras [72]. On binding with GTP, Ras targets downstream effectors to initiate different signals in response to growth cues. In contrast, Ras GTPase activating proteins (RasGAPs) encoded by tumor-suppressor genes (*p120GAP* and *neurofibromin 1*) can catalyze GTP hydrogenation and maintain Ras in the inactive GDP-bound state [73].

**Figure 2:**
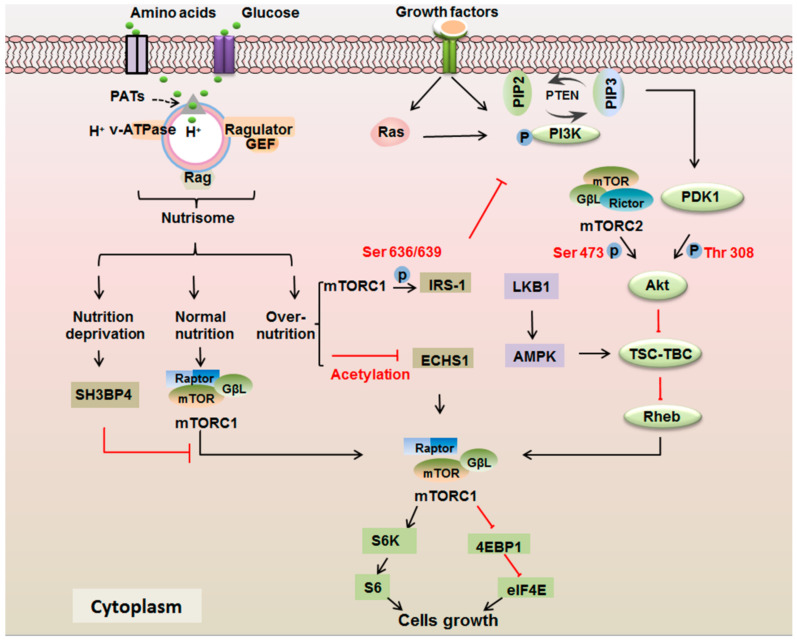
The response model of Ras/phosphatidylinositol 3-kinase (PI3K)/Akt/mTOR pathway to the growth factors, amino acids and glucose levels. mTORC1 and mTORC2 are the two distinct multiprotein complexes of mTOR in function and biochemistry. The mTORC1 complex consists of mTOR, GβL and raptor. The mTORC2 complex is comprised of mTOR, GβL and rictor. The two mTOR complexes have a central role in different kinds of environmental stimulations. Upon the growth factors response, the bindings of ligands and receptors activate Ras and PI3K. PI3K can be activated by both receptor and activated Ras. Active PI3K transfers phosphate groups to phosphatidylinositol 4, 5-bisphosphate (PIP2) and generate phosphatidylinositol 3, 4, 5-triphosphate (PIP3) to recruit PDK1 (phosphorylates Akt on Thr 308) and mTORC2 (phosphorylates Akt on Ser 473). The Akt suppresses TSC-TBC1D7 (TSC-TBC) and promotes Rheb to activate mTORC1 to regulate cell growth by targeting ribosomal S6 protein (S6K1) and eukaryotic translational initiation factor eIF4E-binding protein 1 (4E-BP1). Under the normal nutrition, the amino acids and glucose are absorbed by a nutrisome complex that includes proton-assisted SLC36 AA transporters (PATs), Ras-related GTPase (Rag), Ragulator and v-ATPase to activate the mTORC1. Nutrition deprivation negatively mediates the activation of mTORC1 through the binding between domain binding protein 4 (SH3BP4) and Rag. In the case of over-activation of nutrition, inactive Enoyl-CoA hydratase 1 (ECHS1) by acetylation activates mTOR. There is a negative feedback regulatory mechanism to maintain nutrition balance. mTORC1 can cause inhibition of insulin receptor substrate-1 (IRS-1) Ser 636/639 phosphorylation under over-nutrition, and further restrain PI3K/Akt signaling. The liver kinase B1 (LKB1)/AMP-activated protein kinase (AMPK) pathway is a negative regulator of mTORC1.

PI3K has been shown to be the substrate of active Ras and RTKs (Figure 2). It is comprised of an 85 kDa regulatory subunit and a 110 kDa catalytic subunit. The regulatory subunit binds with receptor via the SH2 domain and catalytic subunit transfers phosphate groups to phosphatidylinositol 4,5-bisphosphate (PIP2) and converts it into phosphatidylinositol 3,4,5-triphosphate (PIP3) [74]. This process is reversed by phosphatase and tensin homologue (PTEN) [75]. Then, PIP3 recruits 3-phosphoinositide-dependent kinase 1 (PDK1) to phosphorylate Akt on Thr 308. Meanwhile, mTORC2 is in charge of Akt Ser 473 phosphorylation [76]. Fully activated Akt inhibits the activity of the TSC–TBC1D7 complex and thereby promotes the GTPase activity of Ras homolog enriched in brain (Rheb), a small GTPase of the Ras family [77]. Tuberous sclerosis complex 1 (TSC1) is a tumor suppressor that combines with tuberous sclerosis complex 2 (TSC2) to block the catalytic Rheb–GTPase-activating protein (GAP) activity of TSC2. Another function of TSC1 is exhibited in the regulation of stability and position of TSC–TBC1D7 [78]. TBC1D7 has been identified as a stably associated and ubiquitous subunit of the TSC1–TSC2 complex [79]. Effective Rheb activates mTORC1 with a four-way interface by binding to the amino-terminal portions of the N-heat, M-heat and FAT domains of mTORC1 [80]. Activated mTORC1 phosphorylates eukaryotic translational initiation factor eIF4E-binding protein 1 (4E-BP1) and ribosomal S6 protein (S6K1) by raptor interacting with the Tor signaling sequence (TOS) motif [81]. The phosphorylation of 4E-BP1 relieves the bond with eIF4E, releasing eIF4E. Meanwhile, active S6K leads to the phosphorylation of ribosomal S6 protein (S6) [82]. The release of eIF4E and activation of S6 has been identified to enhance the mRNAs translation involved in protein synthesis, ribosomal biogenesis and mitochondrial biogenesis [70]. Translation initiation factor 2 is also found to be phosphorylated by mTOR and ribosomal protein S6 kinase (p70S6K) to promote protein synthesis [83].

### 3.2. mTOR Signaling Responds to the Nutrients

In addition to responding to growth factors, mTORC1 signaling also has a critical role in the absorption of nutrients, especially amino acids (AA) (Figure 2). Under normal nutrient stimulation, there is a “nutrisome” complex located in the lysosome and consisting of Ras-related GTPase (Rag), proton-assisted SLC36 AA transporters (PATs), Ragulator and v-ATPase to activate the mTORC1 [84,85]. The key functional molecule is Rag which shuttles mTORC1 to the lysosomal surface, promoting the interaction of mTORC1 and Rheb [86]. There are four Rag members (RagA/B/C/D) in mammals. RagA and RagB equipped GTP constitute a heterodimer with RagC or RagD which is loaded with GDP. This tetramer accomplishes the exchange of GTP and GDP [87]. The transport of amino acids from the lysosome is accomplished by the PATs [88]. “Ragulator’’ is a complex composed of five small proteins—p18, p14, MP1, C7orf59 and HBXIP—and owns GEF activity [89]. The v-ATPase functions as the proton pump of lysosome, supporting proton cycling requirement for the transfer of AAs via PAT1 [90]. The interaction of mTORC1 and Rheb forms a common signal in response to growth factors and AAs in controlling cell growth. Besides Rheb and Rags, the small GTPases RalA, Rab5 and Arf1 have been implicated as playing a role in AA mTORC1 activation [86]. In addition, glucose can activate mTORC1 by the Rag, v-ATPase and regulator complex on the lysosome, implicating the same mechanism in amino acids and glucose regulation by mTOR [85].

Recent research has indicated that mTOR is involved in the nutrient-dependent metabolic checkpoints of mammalian G_1_ cell arrest. Essential amino acids and glutamine contribute to the growth factor-mediated restriction point to enter the S phase from G_1_ phase. Growth factor deprivation decreased the phosphorylation of p70S6K and 4E-BP1. In contrast, essential amino acids and glutamine deprivation had no effect on Akt phosphorylation at either Thr 308 or Ser 473, indicating no effect on PI3K or mTORC2 activity. This indicates that mTOR functions in different pathways in response to growth factors and AA [91]. Abnormal nutrient stimulations such as nutrient deprivation or overabundance of nutrients create stresses on cell growth. Under AA deprivation, SH3 domain binding protein 4 (SH3BP4) is bound to the inactive Rag complex through the SRC homologous 3 (SH3) domain and negatively mediates the activation of mTORC1 [92]. The prevention of mTOR over-activation is key to maintaining nutritional homeostasis [93]. Enoyl-CoA hydratase 1 (ECHS1), a converging enzyme of oxidation of both fatty acids and branched-chain amino acids, localizes at mitochondria and is negatively regulated by over-nutrients. The over-nutrient-induced Lys^101^ acetylation of ECHS1 mediated by amino acid synthesis 5 could inactivate ECHS1. The further ubiquitination of ECHS1 by SH3-domain-containing RING finger protein prevents this from translocating to the mitochondria, causing apoptosis resistance [94]. Remarkably, there exists a negative feedback regulatory mechanism that controls the over-absorption of nutrients. The hyperactivation of the mTOR pathway by hyperglycemia in diabetes can cause insulin resistance due to the desensitization event caused by IRS-1 Ser 636/639 phosphorylation [95]. The mutation of Ser 636/639 totally recovering insulin-stimulated PI3K activity demonstrates that Ser 636/639 phosphorylation negatively controls PI3K. Interestingly, mTOR inhibition by rapamycin or the absence of the C-terminus of raptor significantly suppresses IRS-1 Ser 636/639 phosphorylation and increases insulin signaling via the PI3K/Akt pathway, indicating that mTOR carries out the Ser 636/639 phosphorylation of IRS-1 in a raptor-dependent manner [95]. Therefore, mTOR phosphorylates IRS-1 Ser 636/639 to control increasing concentrations of glucose in an insulin-independent manner and suppresses IRS-1 associated PI3K/Akt signaling. This may be associated with another finding that nutrients affect mTOR–raptor interaction via the nutrient-sensitive element GβL in the mTORC1 complex [96].

The levels of nutrients and energy are also sensed and relayed to mTORC1 by the liver kinase B1 (LKB1)/AMP-activated protein kinase (AMPK) pathway [97]. The upstream phosphorylation of AMPK at Thr 172 is accomplished by LKB1. The pseudo kinase STE20-related adaptor protein and the adaptor protein mouse protein 25 contribute to the activation of LKB1 [98]. Activated AMPK is shown to phosphorylate TSC2 to inhibit mTORC1 signaling. However, TSC2-deficient cells still respond to the inhibition of AMPK-dependent mTORC1 by AMPK activators [99]. In addition to inhibiting mTORC1 by TSC2, AMPK also inhibits mTORC1 activation by the direct phosphorylation of raptor [97].

## 4. The Interplay between mTOR Signaling and the Caspase Family

### 4.1. Impact of mTOR Signaling on the Caspase Family

**Figure 3:**
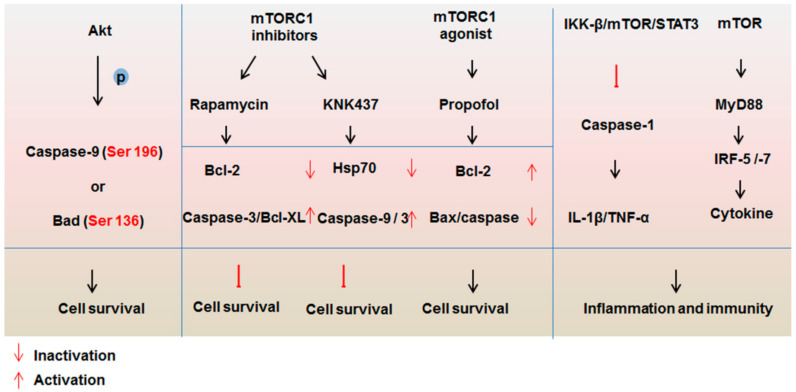
Regulation of mTOR signaling for the caspase family. mTOR signal transduction mediates the caspase family in terms of cell survival and inflammation in a direct and indirect manner. Akt can directly phosphorylate caspase-9 (Ser 196) and Bad (Ser 136), resulting in the limiting of cell survival. The function mechanisms of inhibitors (rapamycin and KNK437) and agonist (propofol) of mTOR indicate that the Bcl-2 family play roles as connectors of mTOR signaling and caspases. In addition, mTOR can inhibit the activation of caspase-1 and mediate the transcription of cytokine via MyD88/IRF-5/IRF-7 to affect inflammation and immunity.

Recent data have implied that mTOR signaling could directly or indirectly affect the stress response mediated by the caspase family (Figure 3). mTOR is found to coordinate the defense process of the immune system by suppressing the vitality of caspase-1 by the I kappa B kinase (IKK-β)/mTOR/signal transducer and activator of transcription 3 (STAT3) axis and regulates the transcription of cytokine-and type I interferon regulatory factor (IRF)-genes through MyD88/IRF-5/IRF-7 pathway [8,100,101]. The inactivation of mTOR inhibited by rapamycin triggers a cytopathic effect and further induces apoptosis after coxsackie virus B3 infection [102]. Rapamycin significantly induces apoptosis by triggering caspase-3 activation, a decrease of Bcl-2 protein expression and the upregulation of pro-apoptotic factor Bcl-XL [103,104]. N-formyl-3, 4-methylenedioxy-benzylidene-c-butyrolaetam (KNK437), one of the derivatives of rapamycin, was found to disrupt the mTOR–raptor interaction on the outer membrane of the mitochondria and inhibit the phosphorylation of mTOR (Ser 2448) and S6K (Thr 389) and trigger caspase-9 and -3 activation by blocking the expression of Hsp70 [105]. The phosphorylation of p70S6K or expression of caspase-3 as surrogate molecular markers serves as the function of monitoring the biological effects of rapamycin derivatives [105]. In contrast, propofol upregulates the protein expression of Bcl-2 and activates mTOR, resulting in the reduction of expression levels of Bax and caspase activation for apoptosis inhibition after hypoxia/reoxygenation treatment to protect the brain from cerebral ischemic–reperfusion injury [106]. These results suggest that the anti-apoptotic and pro-apoptotic factors of the Bcl-2 family play roles as the connectors of mTOR signaling and caspases in determining the fate of cells.

As the upstream regulator of mTOR, Akt plays a key role in many cell growth processes, such as glucose metabolism, apoptosis, cell proliferation, transcription and cell migration. The substrates of Akt are wide-ranging, including molecules regulating cell survival (such as the caspase family, death receptor, the pro-survival Bcl-2 family members, FOXO transcription factors, NF-κB transcription factor and p53), cell proliferation and cell growth (p21, p27, mTORC1, TSC2, glycogen synthase kinase-3 and prolin-rich Akt substrate of 40 kD), angiogenesis (endothelial nitric oxide synthase, hypoxia-inducible factor 1-alpha and hypoxia-inducible factor 2-alpha transcription factors) cellular metabolism (glucose transporter 4) and cell migration and invasion (nuclear factor of activated T cells transcription factors) [107,108,109]. Akt may directly phosphorylate the downstream substrates or indirectly control these regulatory points by changing the gene expression level. Among the caspase family, caspase-9 is the only member that has been shown to be directly phosphorylated by Akt. The phosphorylation is located at Ser 196 [110]. This may be a cell protection method when a certain degree of apoptosis has occurred. For example, Akt can phosphorylate caspase-9 and lead to the inhibition of cell apoptosis partially when some quantity of cytochrome c has been released from mitochondria [110]. Bad can be phosphorylated by Akt on Ser 136. The absence of this phosphorylation could inactivate the pro-survival protein Bcl-XL, inducing the release of cytochrome c and causing cell death [111]. However, other research demonstrates that Akt could suppress the release of cytochrome c irrespective of whether the cells express Bad [112].

### 4.2. Protease Properties of Caspases are Important for mTOR Signaling

The prominent role of caspases in regulating mTOR signal transduction is due to the protease properties. The catalytic activity of caspase is based on the evolutionarily conserved Cys sequence and maintains a highly specific cleavage for substrates at the terminal of carboxy of Asp residues [10]. For this reason, these proteins were named as caspases. Scanning the active site of caspase for synthetic substrates and endogenous substrates is helpful to investigate the biological significance of this type of enzyme. Using the positional scanning substrate combinatorial library technique, the WEHD↓ amino acid sequence as the most optimal recognition motif for caspase-1 was observed [113]. The fluorescence-activated cell sorting approach revealed that the DEVD↓ sequence was the specific catalytic site of caspase-3 [10]. Caspase-8 has a predilection on the VEXD↓ or LEXD↓ sequence [114]. Although caspases, as the crucial apoptosis proteins, cleave their substrates at the Asp site, for example, caspase-3 has almost absolute demand for Asp residues [115,116], motifs containing glutamate or phosphoserine residues recognition sites are also identified to be active sites of caspases [117]. Kinetic analyses of caspases-3 and -7 show that the recognition motif DEVD↓ of substrates are cracked only two-fold higher than the DEVE↓ sequence. Only caspase-3 acts on the protein chains comprising the DEVpS↓ sequence. With the finding of the amplified identified substrates of caspases, this shows that other than the specific sequences, the structure and extended sequence of caspase substrates can explain the cleaved preferences of caspases [10]. For instance, caspase-7 has higher potential for poly ADP-ribose polymerase cleavage than caspase-3 using exosites [118].

**Figure 4:**
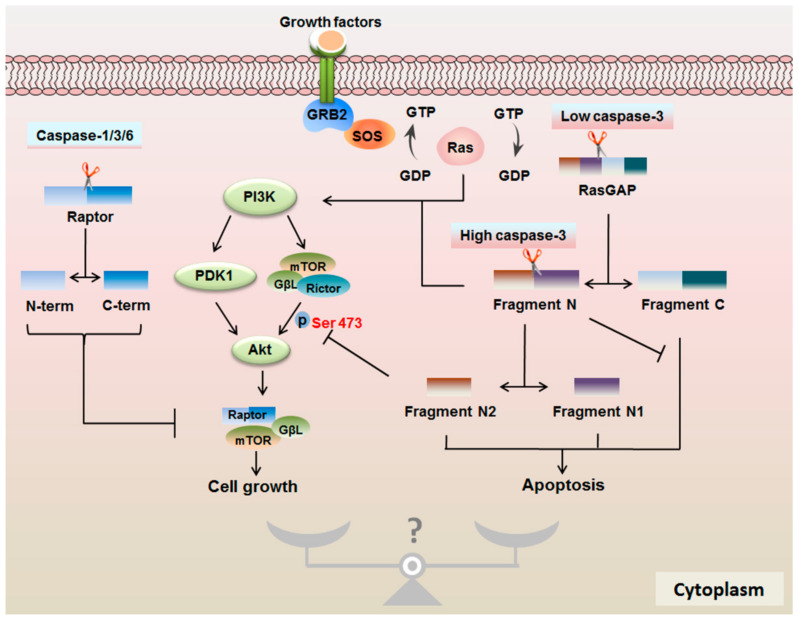
The new model of apoptosis regulated by the caspase-dependent cleavage of mTOR and Ras GTPase-activating protein 1 (RasGAP). Human raptor can be cleaved into N-terminus and C-terminus by active recombinant caspase-1, -3 and -6 in vitro. This cleavage of raptor is similar to the inhibition mechanism induced by rapamycin, which disturbs the interaction of raptor and mTOR. The binding of growth factors and receptor recruits adaptor proteins containing the SRC homology domain 2 (SH2) or phosphotyrosine binding domains such as growth factor receptor-bound protein 2 (GRB2). Son of seven less homolog protein (SOS) is subsequently activated and stimulates the exchange of GDP for GTP on Ras. In contrast, RasGAP catalyzes GTP hydrogenation and maintains Ras in the inactive GDP-bound state. The cleavage sites of RasGAP induced by caspase-3 contain 455 aspartic acid and 157 aspartic acid position. The low activity of caspase-3 cleaves the RasGAP into fragment N and fragment C at region 455. Fragment N can achieve cell protection, and fragment C induces apoptosis, which is restrained by fragment N. With the increasing of caspase-3 activity, site 157 is cleaved and generates fragment N1 and fragment N2, which contribute to cell death by blocking the phosphorylation of Akt at Ser 473. This is a new model for the regulation of apoptosis.

The endogenous substrates of caspases are involved in apoptosis events and the more extensive non-apoptotic function, increasing the biologically significant of caspases. For instance, there is a novel growth-controlling mechanism involving caspase cleaving a specific active site on the substrates, which regulates growth. The component of mTORC1, raptor, is found to be the target of caspases. Raptor binds to mTOR via both N- and C-terminal regions and has a caspase-like domain, allowing it to play the pivotal protease property in the interaction with 4E-BP1 and p70S6K1 via TOS motifs. TOS motifs are strongly similar to many caspase recognition sites [119]. The interaction of raptor–mTOR is the central structure for mTORC1. The classical inhibitor of mTORC1 rapamycin binds to FKBP12 and the FRB domain of mTOR to inhibit mTORC1 allosterically. Other than rapamycin, R Martin found that the inhibitors of mTORC1, etoposide and cisplatin, functions an anti-cancer effect due to inducing the cleavage of raptor, disrupting the binding of raptor–mTOR [120]. Noticeably, the proteolytic activity of caspase on raptor has an irreversible effect on mTORC1 function (Figure 4). Human raptor can be cleaved into 100 kDa and 70–80 kDa by active recombinant caspase-1, caspase-6 and caspase-3, but not caspase-7 in Jurkat T-cell lysates. The cleavage site induced by caspase-6 exists at two sites in three conserved domains of raptor (a raptor N-terminal-conserved domain, three HEAT repeats and seven WD repeats). These domains affect raptor–mTOR binding [60]. One caspase-6 cleavage site is located in DDADD residues of C-terminal part of raptor. Another active site is at the N-terminal part after DEADLTD residues. The latter could block the interaction of 4E-BP1 and S6K with raptor because the N-terminus of raptor is exposed to the outside, and it is easier to access 4E-BP1 and S6K [120]. Further evidence demonstrates that TRAIL could cleave raptor and rictor in a caspase-dependent manner [121]. This research provides a new mechanism for caspase regulating cell death by cleaving raptor or rictor to inactivate the two mTOR complexes. Not all caspases can perform the cleavage of mTORC1 complex. In addition to raptor, the degradation of Cbl, Cbl-b, Raf-1, Akt-1 and Ras GTPase-activating protein 1 (RasGAP, also named p120GAP) have been found to be dependent on caspase activation during Fas-induced apoptosis [122]. Similar to raptor, the degradation of Raf-1 and Akt-1 by caspase inhibits their kinase activity. In the Ras/PI3K/Akt/mTOR pathway, Cbl is an adapter molecule and interacts with PI3K [123]. However, of these proteins, RasGAP is the sole directly cleaved protein by caspase in vitro, indicating that RasGAP is a DEVD-active downstream of caspase upon stress response.

### 4.3. RasGAP Controls the Cell Fate in a Caspase-3 Activity-Dependent Manner

An important cell growth sensor here is RasGAP. Caspase-3 is required for RasGAP cleavage in response to different stresses and thus determines the fate of cells (Figure 4). As mentioned above, in the pathway of Ras/PI3K/Akt/mTOR, RasGAP which is tumor suppressor gene acts as GTPase-activating proteins to catalyze GTP hydrogenation with GAP-related catalytic domain and maintain Ras in the inactive GDP-bound state [73]. N-terminal region of RasGAP contains two SH2 domains and a single SH3 domain. The C-terminal region contains a pleckstrin homology (PH) domain, two C2 domains and the RasGAP domain [73]. The SH2 domains, as functional phosphotyrosine sites, can interact with active receptors and specific phosphoproteins [124]. For example, upon growth factors stimulation, the N-terminal SH2 domains of full RasGAP bind to fibroblast growth factor receptor (FGFR) and recruit Akt to the new complex, forming a RasGAP/Akt/receptor complex [125]. Akt bears the PH domain associated with the membrane for activation [126]. The GRD, located at residue regions 702–1044 of the GAP C-terminus, catalyzes GTP hydrogenation, which is mediated by the N-terminus [127]. Once recruited by RTK, RasGAP promotes the GTPase activity of Ras via the GAP domain [128]. This is a negative regulation of Ras activation [73].

Although it seems that RasGAP is a negative regulator of the Ras/PI3K/Akt/mTOR pathway, RasGAP can also promote growth factor-regulated cell growth with a different mechanism. The full length of RasGAP can be cleaved into segments with different functions by caspase-3 (Figure 4). RasGAP contains 1047 amino acid sequences bearing two cleaved sites: position 455 aspartic acid and 157 aspartic acid executed by caspase-3 [129]. It has been shown that caspase-3 with low activity can first cleave off fragment N and fragment C at the DTVD↓ 455G region of RasGAP. Fragment N and fragment C are about 56 kDa and 64 kDa, respectively [9]. It has been found that single fragment C can induce apoptosis [9] and has the function of activating caspase-3 and cleaving poly ADP-ribose polymerase. However, this function could be completely reversed by N-terminal fragments [9]. Further research indicates that apoptosis triggered by fragment C works in a caspase-dependent manner. In contrast, fragment N contributes to cell protection through the Ras/PI3K/Akt/mTOR pathway. Data reveal that fragment N could increase insulin-induced ERK2 phosphorylation and G_2_/M transition through interacting with RhoGAP via the SH2 domain to promote cell growth [130].

The increasing activity of caspase-3 continues to cleave RasGAP at the DEGD↓ 157S sequence in fragment N and produces two smaller N-terminal fragments, called fragment N1 and N2 [131]. Fragment N is necessary for the production of fragment N2 due to the mutation of fragment N does not induce the production of fragment N2 with high activity of caspase-3 [129]. The fact is that the first cleavage of RasGAP supports the cleavage of 157 S sequence sites for structural modifications [9]. Fragment N1 and N2 are associated with apoptosis. Single fragment N1 and fragment N2 increase the sensitization of cells to apoptosis triggered by cisplatin, but this effect does not include fragment C [9]. In contrast to fragment N, fragment N2 significantly restrains ERK phosphorylation and the G_2_/M transition. Importantly, it directly suppresses the phosphorylation of Akt Ser 473, further attenuating the phosphorylation of the Thr 308 and leading to a decrease of Akt activity. Finally, it serves as an apoptosis inducer [130].

What kind of mechanism will make fragment N and its two sub-segments produce an opposite function? Further research demonstrates that the sole segment of SH2–SH3–SH2 can also block the ERK phosphorylation, the G_2_/M transition and the phosphorylation of Akt Ser 473, indicating that the SH2–SH3–SH2 fragment of fragment N2 is the key function structure. In fact, the SH2-SH3-SH2 fragment of fragment N2 interacts with the growth factor receptor, wrecking the existing combination of the Akt/FGFR/RasGAP complex [130]. The SH3 domain alone has no effect on Akt phosphorylation. Although fragment N also has an SH2–SH3–SH2 fragment, it cannot combine with the FGFR complex and thus cannot grab RasGAP and Akt. A hypothesis states that the interaction between the SH3 domain and the polyproline-rich domain of fragment N affects the conformation of the SH2–SH3–SH2 domain and binding with receptor complex. Disrupting the interaction of SH3 domain and the polyproline-rich domain with a mutation of SH3 domain leads to fragment N combining with Akt and the receptor to prevent binding with full-length RasGAP [130]. This result leads to the inhibition of Akt Ser 473 phosphorylation. With the increasing activation of caspase-3, the production of fragment N2 enhances the competition of the SH2–SH3–SH2 domain with Akt and thus inactivates the anti-apoptotic function of fragment N. The anti-apoptotic function of fragment N and pro-apoptosis function of fragment N2 indicate that Akt Ser 473 phosphorylation triggered by mTORC2 is the key regulation mechanism for cell fate. All N-terminal RasGAP fragments can suppress fragment C-induced apoptosis, suggesting that they may have a common structure which could disturb the action of fragment C or that could indirectly restrain fragment C-induced apoptosis via other anti-apoptotic signals.

The fragment N2 induced-apoptosis forces us to focus on the biological function of Akt. The recruiting of Akt to the plasma membrane supports the assumption that Akt has an optimal conformational change for Akt activation [107]. The translocation of Akt can be carried out via PI3K-generated phospholipids directly or phospholipids-mediated kinases such as the mTORC2 or PDK-1 [107]. Akt has three distinct isoforms: Akt1, Akt2 and Akt3 [132]. Research suggests that Akt1 plays an irreplaceable role in the response to RasGAP-driven fragment compared with other two isoforms. This is because the two activated phosphorylated residues Thr 308 and Ser 473 both exist in Akt1 [133]. The phosphorylated Thr residues of two other isoforms include Thr 309 in Akt2 and Thr 305 in Akt3 by PDK1. Phosphorylated Ser residues contain Ser 474 in Akt2 and Ser 472 in Akt3 by mTORC2 [134,135,136]. It has been demonstrated that the phosphorylation at Ser 473 of Akt is located upstream of the phosphorylation at the Thr 308 and promotes it. Therefore, the phosphorylation on Ser 473 of Akt is crucial for the function of Akt. Blocking this phosphorylation site can activate pro-apoptotic substrates of Akt and inhibit mTORC1, resulting in apoptosis. Fragment N-mediated cell protection in a PI3K-dependent manner is regulated by blocking apoptosis. Akt not only inactivates pro-apoptosis proteins but also activates anti-apoptotic factors to regulate cell survival. The cleavage of RasGAP by caspase-3-generated fragments can play different effects with an Akt-dependent way. The inhibition of mTORC1 with rapamycin or the silencing of raptor has no effect on the cell protection of fragment N [137]. In fact, fragment N could suppress the activation of NF-κB against inflammatory stimulation [127,138]. The absence of Bad also displays a protective function of fragment N against cisplatin-induced death, indicating that the Ser 136 phosphorylation of Bad by Akt partially promotes fragment N-mediated protection and is not necessary [137]. In addition, Akt can play anti-apoptotic effects by directly phosphorylating Ser 196 on human pro-caspase-9 in vitro, but this is restricted in mammals [110]. It seems to be a universal blocker of caspases-induced apoptosis because it inhibits caspase-9-induced cell apoptosis [139]. Three N-terminal fragments of RasGAP could block the apoptosis triggered by low levels of caspase-9, and fragment N1 and N2 invigorate high levels of caspase-9-induced apoptosis. It demonstrates that the full cleavage of RasGAP allows the efficient processing and activation of caspase-3 to induce apoptosis [9]. Remarkably, the inhibition or absence of caspase-3 is defective in activating the anti-apoptotic Akt kinase in response to various environments with an increase of cell death and impaired survival in some cases [140]. A similar defect is found in RasGAP-mutated mice. It seems to demonstrate that caspase-3 is a double-sided effector.

Fragment N achieves cell protection by Akt-mediated mTORC1, Bad, NF-κB or caspase-9, explaining why cells with mildly activated caspase-3 do not necessarily experience death. Fragment N provides a selective survival advantage for cells. In fact, fragment N2 promotes cell to die and increases the susceptibility of tumor cells to genotoxins [129]. This phenomenon is not found in normal cells. Therefore, RasGAP as a stress sensor responds to caspase activity and decides the fate of cells. The anti to pro-apoptotic nature of RasGAP ensures cell survival when cells undergo different stresses and also supports the new apoptotic model. In this process, caspase and mTOR signaling are integrated and coordinated to determine the fate of cells.

## 5. Conclusions

Although the role of mTOR signal transduction and the caspase family has been widely studied, the combined effect of the mTOR signaling and the caspase family requires deeper exploration. We now know that they can affect each other in a direct or indirect manner. They both contribute to the decision of a cell’s fate upon complex environmental cues and stresses. Importantly, the data support a novel cell protection and apoptosis model. Despite the related researches having elaborated the mechanism behind this, outstanding questions remain to be answered. For example, in many of the stress sensors for decision-making cell survival such as p53, caspase and RasGAP, who is the final decision maker? What is the role and mechanism of the caspase-dependent cleavage of raptor in the Ras/PI3K/Akt/mTOR pathway response? Are there other caspase-dependent active substrates? Fragment N2 could increase the susceptibility of tumor cells to the genotoxins, implying that it could be applied in the tumor therapy. In summary, the caspase-dependent cleavage of mTOR signaling and the effect of mTOR on caspases constitute a complex and huge network in response to stresses, supporting a new therapeutic strategy for disease.

## Data Availability

Not applicable.
